# The effect of tobacco expenditure on expenditure shares in South African households: A genetic matching approach

**DOI:** 10.1371/journal.pone.0222000

**Published:** 2019-09-06

**Authors:** Grieve Chelwa, Steven F. Koch

**Affiliations:** 1 Graduate School of Business, University of Cape Town, Cape Town, South Africa; 2 Department of Economics, University of Pretoria, Pretoria, South Africa; National Institute of Public Finance and Policy, INDIA

## Abstract

This paper examines whether tobacco expenditure leads to the crowding out or crowding in of different expenditure items in South Africa. We apply genetic matching to expenditure quartiles of the 2010/2011 South African Income and Expenditure Survey. Genetic matching is a more appealing approach for dealing with the endogeneity of tobacco expenditure that often plagues studies using systems of demand equations. Further, genetic matching provides transparent measures of covariate balance giving the analyst objective means of assessing match success. We find that the poorest tobacco consuming households in South Africa consistently allocate smaller budget shares towards food items than non-smoking households. Specifically, we find that dairy, fruits, nuts and oils are displaced in favour of tobacco expenditure in the two poorest quartiles. Unsurprisingly, food items are never displaced for households in the top two quartiles, given these households’ greater access to resources. Like other studies in the literature, we find that tobacco expenditure consistently crowds-in alcohol across all quartiles confirming the strong complementarities between the two.

## Introduction

According to economic theory, consumer behaviour is best understood within the confines of a demand system. Demand systems rely upon the accessibility of price data, among other data requirements, for reliable estimation. In particular, price data allow for the estimation of cross-price elasticities and these elasticities determine the substitutability or complementarity of various goods. In the case of tobacco products, a statistically significant positive cross-price elasticity between, say, tobacco and food implies that food expenditure can be crowded-out by tobacco expenditure because increases in tobacco prices increase the demand for food [1]. It can be difficult to access the depth of price data needed for the estimation of cross-price elasticities in many low- and middle-income countries (LMICs). Therefore, researchers working on these countries have adopted different approaches to analyzing the effect of tobacco on household expenditure behaviour.

One early example finds that in Bangladesh, cigarette consumption worsens living standards while in rural households tobacco expenditure is larger, as a share of the weekly budget, than it is for vegetables and milk [2]. Implicit in the Bangladesh analysis is an assumption that tobacco expenditure crowds-out expenditure on other goods in the same proportion as total expenditure is allocated. This may not be true because expenditure decisions are part of a system and should be treated as such. Placing tobacco expenditure within a conditional demand system reveals the potential for non-proportional response as well as underpinning a statistical test of differences in preferences between tobacco-consuming and non-consuming households [3].

Subsequent work has, therefore, applied variations of the aforementioned conditional demand system in India, Taiwan, South Africa, Cambodia, Zambia, Turkey, Mauritius and Bangladesh [4–12]. Much of this work does find that tobacco expenditure crowds out expenditure on different types of food particularly among poorer and the rural population. Additionally, this work finds that tobacco crowds-in alcohol confirming the hypothesized complementarities between the two. Beyond these two commodity groups, there is much heterogeneity in results across countries and studies.

Country heterogeneity in results might be explained by differences in tobacco control regimes across countries. In Mauritius, for example, expenditure allocations were different before and after the imposition of a series of tobacco control measures in the period 2009 to 2012 [11]. In Turkey, on the other hand, there was little evidence that tobacco control policies mattered for expenditure allocations [10].

The differences across countries might also be explained by the inability to account for the endogeneity of tobacco expenditure in conditional demand systems [8]. A common instrumental variable used in the literature in LMICs is the adult sex ratio. This is because male smoking prevalence is much higher than female smoking prevalence in many LMICs [4]. However, the gender make-up of the household is likely to also explain other household consumption expenditure decisions given that men and women generally have different preferences. In other words, the adult sex ratio is an imperfect instrument and possibly a bad instrument. If one is willing to assume the direction of the bias that might arise from the adult sex ratio as an instrument, as has been done by Chelwa and van Walbeek (2014), it is possible to bound the crowding-out/crowding in estimates using the method proposed by Nevo and Rosen (2012) [8, 13]. However, these assumptions are not testable such that the bounds may not contain as much information as is suggested. Other instrumental variables used in the literature, like measures of local or regional smoking prevalence [6], are likely to suffer from similar shortcomings as the adult sex ratio given that smoking prevalence also has a gender dimension.

The previous literature focuses on the effect of tobacco consumption on household expenditure and, therefore, the original conditional demand model is often simplified to include a binary indicator of tobacco expenditure and an interaction term with net household expenditure [7, 8, 11]. In this paper, rather than assuming that the adult sex ratio (or any other instrument) is imperfect, we assume that it is one of a number of factors (like education and income, among others) that underscores household tobacco consumption decisions. Furthermore, rather than directly estimating the binary tobacco indicator within a conditional demand system we test for crowding out using the genetic matching approach proposed by Diamond and Sekhon (2013) [14]. Genetic matching is an approach to multivariate matching that uses a genetic algorithm (i.e. a search algorithm) to optimize covariate balance between treated and untreated units in observational studies. The algorithm iteratively searches over a space of distance metrics in search of metrics or combinations of metrics that best match covariates between the two groups. In addition, the algorithm assigns relatively bigger weights to covariates that contribute the most towards improving balance between treated and untreated units. The technical details of genetic matching are discussed in the next section.

For a host of reasons, we believe that genetic matching is a more credible method for investigating the causal relationship between tobacco expenditure and household consumption decisions. First, it does not require an instrumental variable satisfying the rarely, if ever, met exclusion restriction. Second, the assumptions required under genetic matching are much less stringent than those for instrumental variables (IV) regressions. Third, genetic matching is nonparametric which offers improvements in two dimensions. It does not impose linearity on the relationship between the control variables and our outcome of interest, as is expected in both ordinary least squares (OLS) and IV regressions. Therefore, the approach allows the data to “speak for itself”. Fourth, genetic matching contains within it as special cases the widely popular propensity score matching and Mahalanobis distance matching [15, 16]. Matching on the propensity score requires the correct specification of the propensity score function, something that is rarely satisfied in practice [14]. Unlike matching on the Mahalanobis distance, the genetic matching algorithm searches over a range of distance metrics to find one that optimizes covariate balance. Such a metric may or may not be the Mahalanobis distance function, but this is not assured *a priori*. Lastly, genetic matching transparently provides measures of covariate balance giving the practitioner an objective assessment of whether matching has been successful or not.

Using genetic matching across expenditure quartiles, we find that the poorest tobacco consuming households in South Africa (the bottom two quartiles) systematically allocate smaller budget shares towards some food categories than non-smoking households. Specifically, we find that dairy, fruits and nuts and oils are displaced in favour of tobacco expenditure in the two poorest quartiles. Unsurprisingly, these items are not displaced for households in the top two quartiles, given these households’ greater access to resources. We also uncover instances of the crowding-in of “luxury” items only among well-off households. For example, gambling and restaurant/hotel expenditures are consistently crowded-in in the top 2 quartiles. This again speaks to the fact that budget constraints are less binding for well-off households. Like many of the studies in this literature, we find that alcohol expenditure is consistently crowded-in in all 4 quartiles. The additional expenditure share given to alcohol ranges between 1 and 4 percentage points in South African households.

The rest of the paper is structured as follows. Section 2 presents the method, Section 3 discusses the data, Section 4 presents the results while Section 5 discusses them. Section 6 concludes.

## Method

Our primary interest is the impact of tobacco consumption on household expenditure behaviour. Thus, we denote $T_{i}=I(t_{i}>0)$, i.e., $T_{i}=\{0,1\}$ as an indicator function for whether or not household *i* purchases any tobacco products. In terms of the household budget, tobacco expenditure, *t*_*i*_, is one component of total expenditure (*x*_*i*_). We follow the convention in the literature in defining *M*_*i*_ as household expenditure net of tobacco expenditure, i.e. *M*_*i*_ = *x*_*i*_−*t*_*i*_. Because households differ across a number of dimensions, which are likely to affect their decisions, we denote their characteristics by ***Q***. Although *x*_*i*_ is one of those characteristics, we define it separately for this discussion. A budget share (*w*_*ij*_) of good *j* in household *i* is defined as the ratio of expenditure on the good (*x*_*ij*_) to total household expenditure net of tobacco expenditure, i.e., *w*_*ij*_ = *x*_*ij*_/*M*_*i*_.

The preceding can be used to outline the potential outcomes framework, a framework that we follow in this paper [17]. Define $w_{j}^{0}$ as the potential expenditure share (on good *j*) for households not purchasing tobacco products and $w_{j}^{1}$ as the potential expenditure share for households that do (subscript *i* is removed to prevent notational clutter). From these, we define the average effect of tobacco expenditure on share *j* for households that purchase tobacco as:

$$\tau_{j}=E[w_{j}^{1}|T=1]-E[w_{j}^{0}|T=1]$$ (1)

The first term in (1) is observed in the data whereas the second term is unobserved and must be estimated. The counterfactual second term is the share of the non-tobacco purchasing household budget devoted to good *j*, if that household were instead a tobacco-consuming household. The potential endogeneity of the tobacco indicator, T, is also of concern given that tobacco consumption is unlikely to be randomly assigned across households.

Previous literature has attempted to estimate this effect and deal with endogeneity through instrumentation within a conditional share system, which also includes additional household controls. This system *j*∈{1,*J*} of equations often takes the following form, although other forms exist:

$$w_{ij}=\phi_{1j}+\phi_{2j}T_{i}+Q_{i}\Upsilon_{j}$$ (2)

In (2), potential crowding-out/crowding in of expenditure is defined by the estimate of *ϕ*_2*j*_. If it is negative, tobacco consumption crowds-out that consumption item and crowding-in takes place if the sign is positive. To estimate (2), researchers employ seemingly unrelated regression or three-stage least squares, where the latter incorporates instrumental variables. In addition to the endogeneity of the tobacco indicator, household expenditure, which is often part of the covariate vector ***Q***, is endogenous within a share system. Therefore, at least two instruments are required. The instrument commonly applied to the tobacco indicator, $T_{i}$, is the adult sex ratio, because males are more likely to smoke than females in LMICs [4]. Household smoking prevalence is another possible instrument [6]. However, since this prevalence is underscored by household age and gender structure, it could suffer from limitations also associated with the adult sex ratio.

Statistically, instrumentation requires an exclusion restriction: the instrument cannot explain the expenditure share as well as being able to explain tobacco consumption. Since the age and gender structure of the household are likely to determine preferences in the household, they will also affect consumption decisions and, therefore, expenditure shares within the household. In other words, the exclusion restriction is unlikely to be met. Although relaxing the exclusion restriction and making assumptions about the direction of correlation allows one to bound the instrumental variable estimates, one cannot be certain that the underlying unobserved correlation is, in fact, working in the direction assumed [8].

In our analysis, we take a different approach and assume that conditioning on the observed information addresses the endogeneity. More specifically, we assume strong ignorability. One reason for making this assumption is that we do not believe the adult sex ratio meets the requirements for an instrument, because it is likely to also explain household expenditure decisions. For this study, strong ignorability implies that once we have properly controlled for what we can observe, household tobacco consumption can be assumed to be exogenous, such that instrumentation is no longer necessary.

To explain strong ignorability, in addition to the above variables, we denote ***U*** to refer to unobserved information. Given ***Q***, T and ***U***, strong ignorability or unconfoundedness requires T⫫U|Q. In other words, once we have incorporated the observed information in ***Q***, which includes the adult sex ratio among other variables, tobacco purchase is as good as randomly assigned. This assumption can be operationalized through regression analysis incorporating all of these variables, or through matching. With regression, however, all households are included some of which could be unrepresentative of even tobacco abstainers. For example, if poor adult-only female-only households do not purchase tobacco, while poor adult-only male-only households or adult-only mixed households do, the first group (possibly abstainers) would not be representative of the tobacco purchasing population, and would not be useful for comparison. Therefore, they should not be included in the conditional mean regression model. Furthermore, regression estimates hinge on correct model specification, which also hinges on linearity. Matching, on the other hand, is nonparametric since a model is not specified. A matching estimate is simply a mean difference (as in Eq 1 above). Although there is no guarantee that abstaining households will not be matched or that all matches will be perfect, matching does not incorporate unrepresentative types of households. By not including such households, matching also requires the practitioner to interpret the results with this in mind.

Matching is often determined by a propensity score, although other options exist. Following Rosenbaum and Rubin (1983), a propensity score is given as $e(T_{i})=prob(T_{i}=1|Q_{i})=E[T_{i}|Q_{i}]$ – $Q⫫T_{i}|e(T_{i})$ [15]. In other words, matching on *e* yields distributions of ***Q*** across the tobacco-purchasing and non-purchasing households that are asymptotically equivalent, such that the only difference between matched households is their tobacco purchase behaviour and the implications of this on expenditure shares. However, matching only on the propensity score may not yield matches as good as expected asymptotically because, in practice, we do not know the true propensity score. In other words, practitioners are required to consider a number of alternative propensity score formulations. Furthermore, in testing balance it is common to only consider mean differences, yet household information is often continuous in nature. As such, a simple comparison of means (for the observable variables) between the two types of households may not be enough to assess balance because the mean is only one moment of a distribution.

Therefore, we prefer a generalized version of the propensity score matching algorithm, genetic matching, proposed by Diamond and Sekhon (2013) [14]. Genetic matching is an iterative matching algorithm that incorporates both propensity score and Mahalanobis distance matching [14]. It optimizes balance between the observed covariates depending upon the rule imposed by the practitioner, and it has been shown to perform well in Monte Carlo experiments [18]. As implied, an “optimal balance” metric or loss function is required. In our analysis the loss function minimizes the maximum p-values from Kolmogorov-Smirnov (KS) test statistics (or paired t-tests in the case of discrete variables). The R code used for implementing the genetic matching algorithm in our study is available upon request from the authors.

Diamond and Sekhon (2013) recommend matching on the propensity score in addition to the covariates [14]. Therefore, we estimate (and predict) a propensity score from a logit model based on the household’s structure. In it, we include the household location (province and urban/rural indicators), the age of the household head, the number of household members in various age/gender groups. We also include an indicator of the household head’s exposure to post-school training. In addition to the predicted propensity score, the genetic matching algorithm includes the household head’s schooling level, race and gender indicators of the household head, household income (in natural logarithms), household expenditure net of tobacco (in natural logarithms), the adult sex ratio and the adult ratio. Each of the last four variables are also interacted with the race and gender of the household head. Although we match only on this small set of variables, we test balance across a much wider range of variables and matching is effective (see the Results section and the Supporting information). Due to space concerns, we only report in the Results section a summary of match effectiveness rather than all of the match results. The full set of results are available in S1–S8 Tables and S1–S4 Figs in the Supporting information.

## Data

The analysis in this paper is based on expenditure data from the 2010/11 Income and Expenditure Surveys (IES) conducted by Statistics South Africa [19]. The IES has been conducted quinquennially since 1990. Whereas previous versions of the survey required respondents to recall expenditure either over the past year or the past month, more recent versions incorporate an expenditure diary method. Newer versions of the survey also incorporate the classification of individual consumption by purpose (COICOP) categories. COICOP lists 14 separate categories and each has a number of subcategories. For example, tobacco is contained in subcategory 02.2. In total there are 895 unique consumption items recorded in the data and these are aggregated into broad subsets. In 2010/11 the survey was completed over the course of a year, September through August. Thus, all expenditure data were adjusted to March 2011 using the Consumer Price Index (CPI). The IES underpins the construction of South Africa’s CPI.

In terms of those subsets, categories 01 and 02.1 (food and beverages) are disaggregated into grains, meats, dairy, nuts and oils, fruits, vegetables, sweets, other foods, non-alcoholic beverages and alcoholic beverages. Categories 03, 06–08 and 10–12, form separate categories. Thus, we include clothing, health, transport, communication, education, restaurants and hotels and miscellaneous goods. We also split housing costs (category 04) into two components: one focused on household energy and the other focused on the actual dwelling (“HH Costs” in the tables below). In addition, we separate recreation (09) into two components: gambling and the rest. We refer to the rest as recreation and we refer separately to gambling. Finally, we separate domestic services and cleaning supplies from the furnishing and appliances category (05). We refer to the broad category as furnishings and the separate component as cleaning and domestics.

Although our expenditure aggregation broadly follows the COICOP categorization, we have not followed it entirely because we are interested in whether or not tobacco expenditure had different effects on the consumption of different types of products. In addition, we are interested in comparing our results with those found in the broader literature on the economics of tobacco control.

Descriptive statistics for the two types of households that we study in this paper are presented in Table 1. In the table we present sample descriptive statistics using all data, comparing tobacco purchasing households to those who do not purchase tobacco. For Table 1 we look across the entire sample, focusing our attention on the lack of balance which implies there is extensive potential for improved comparability after matching. For the extended analysis in the Results section, our focus is on expenditure quartile splits of the data and descriptive statistics both before and after matching, are available in the Supplementary information in S1–S8 Tables. Paired t-tests are used for discrete variables and the Kolmogorov-Smirnov tests (ks-tests) are used for continuous variables. Statistical significance is defined at the 5% level. The takeaway from Table 1 and S1, S3, S5 and S7 Tables is that tobacco-purchasing and non-purchasing households are rather different, on average. The differences are particularly stark in household structure.

**Table 1:** Descriptive statistics before matching for the entire sample.

Variable	Non-Smoking Average	Smoking Average	t-probability	ks-probability
Propensity Score	0.222	0.304	0.000	0.000
HH Head Age Group	10.237	10.215	0.632	0.000
HH Head Schooling	1.832	1.692	0.000	0.000
HH Head Training	0.157	0.131	0.000	NA
Black HH Head	0.851	0.673	0.000	NA
Coloured HH Head	0.071	0.232	0.000	NA
White HH Head	0.079	0.095	0.000	NA
Female HH Head	0.516	0.697	0.000	NA
Black HH Log Income	6.801	5.295	0.000	0.000
Coloured HH Log Income	0.617	1.990	0.000	0.000
White HH Log Income	0.778	0.937	0.000	0.000
Female Head Log Income	4.375	5.767	0.000	0.000
Log Net Expenditure	8.189	8.147	0.007	0.000
Black HH Log Net Expenditure	6.810	5.276	0.000	0.000
Coloured HH Log Net Expenditure	0.606	1.945	0.000	0.000
White HH Log Net Expenditure	0.772	0.925	0.000	0.000
Female Head Log Net Expenditure	4.324	5.689	0.000	0.000
Black HH Adult Sex Ratio	0.365	0.408	0.000	0.000
Coloured HH Adult Sex Ratio	0.031	0.113	0.000	0.000
White HH Adult Sex Ratio	0.036	0.047	0.000	0.000
Female Head Adult Sex Ratio	0.329	0.470	0.000	0.000
Black HH Adult Ratio	0.633	0.554	0.000	0.000
Coloured HH Adult Ratio	0.054	0.181	0.000	0.000
White HH Adult Ratio	0.069	0.083	0.000	0.000
Female Head Adult Ratio	0.414	0.587	0.000	0.000
Girls (0–4) in HH	0.209	0.171	0.000	0.000
Boys (0–4) in HH	0.213	0.170	0.000	0.000
Girls (5–14) in HH	0.402	0.303	0.000	0.000
Boys (5–14) in HH	0.403	0.315	0.000	0.000
Women (15–64) in HH	1.233	1.064	0.000	0.000
Men (15–64) in HH	1.001	1.268	0.000	0.000
Women (65+) in HH	0.216	0.186	0.000	0.000
Men (65+) in HH	0.116	0.141	0.000	0.000
Eastern Cape Province	0.090	0.207	0.000	NA
Western Cape Province	0.140	0.114	0.000	NA
Northern Cape Province	0.041	0.072	0.000	NA
Free State Province	0.073	0.136	0.000	NA
Kwa-Zulu Natal Province	0.150	0.081	0.000	NA
Northwest Province	0.102	0.100	0.760	NA
Gauteng Province	0.156	0.144	0.027	NA
Mpumulanga Province	0.097	0.079	0.000	NA
Urban	0.610	0.722	0.000	NA

The table shows relevant descriptive statistics generated from the 2010/2011 South Africa Income and Expenditure Survey (IES) across non-smoking and smoking households before matching. The last two columns are p-values associated with the test that means between the two households are statistically different [paired t-tests for discrete variables and Kolmogorov-Smirnov (ks) statistics for continuous variables]. HH stands for household. HH Head Age Group is a categorical variable that puts household heads into several age groups; HH Head Schooling is a categorical variable that places household heads into several schooling categories and HH Head Training is a categorical variable that places household heads into several training categories. Log Net Expenditure is the natural logarithm of household expenditure net of tobacco expenditure. All other logs are natural logarithms.

## Results

In this section, we present the results of the matching exercise and the main results pertaining to crowding out/crowding in. We begin by demonstrating the success of the matching.

### Matching gains

Table 2 shows the gains in balance from using genetic matching across the 4 expenditure quartiles. The table shows counts of variables that are statistically different from each other when comparing smoking and non-smoking households (more detailed tables on the actual variables compared in the counts by quartile are available in the Supplementary information in S1–S8 Tables). The first two columns compare discrete variables and last two columns are for continuous variables. A total of 42 variables are compared to check for pre- and post-matching balance, including the propensity score and many other variables related to the demographic and social economic status of the household (see Table 1 for a list of these variables). Statistical significance is defined at the 5% level. Paired t-tests are used for discrete variables and the Kolmogorov-Smirnov tests (ks-tests) are used for continuous variables.

**Table 2:** Balancing summary across quartiles.

	Discrete variables		Continuous variables	
	Before	After	Before	After
**Quartile 1**	12	3	23	1
**Quartile 2**	10	4	23	2
**Quartile 3**	12	0	23	0
**Quartile 4**	9	2	22	1

Table shows counts of statistically significant variables across expenditure quartiles for smoking and non-smoking households before and after matching. The first two columns are for discrete variables and the last two columns are for continuous variables. The complete list of variables that are compared in the counts is contained in Table 1(more detailed tables on the actual variables compared in the counts by quartile are available in the Supplementary information in S1–S8 Tables). Statistical significance is judged using t-statistics for discrete variables and Kolmogorov-Smirnov tests (ks-tests) for continuous variables with the level of significance placed at 5%.

As seen in the table, smoking and non-smoking households are different across many variables, as is also evident in Table 1. After matching, far fewer statistically significant differences remain across the two types of households showing the success of the matching exercise. S1–S4 Figs in the Supplementary information also show the success of the matching exercise. The figures show comparisons of density plots of the natural logarithms of household income and household expenditure between smoking and non-smoking households after matching. These plots are nearly identical for the two types of households again showing that the match is a success.

### Main results

The main results of this paper are presented in Tables 3–6 below. In the tables, we present expenditure share differences before and after matching tobacco consuming households to their non-consuming counterparts for each quartile of the expenditure distribution. The results are presented separately for food and non-food items. The first two columns present results for the unmatched sample, while the last two columns present the results for the matched sample. Columns marked “Difference” show the share difference between smoking and non-smoking households–a negative difference shows that smoking households allocate a smaller share than non-smoking households to that particular expenditure item. Our threshold for a statistically significant share difference is 5% and such differences (for the matched sample only) are italicized and highlighted in bold in the tables.

**Table 3:** Food and non-food expenditure share differences for quartile 1.

**FOOD SHARES**					
	Difference	p-value	Difference	p-value
Grains	-1.430	0.000	-0.570	0.151
Meats	1.457	0.000	0.976	0.888
Dairy	-0.298	0.009	***-0*.*838***	0.000
Nuts and Oils	-0.226	0.002	-0.186	0.096
Fruits	-0.179	0.000	***-0*.*087***	0.045
Vegetables	-0.355	0.005	-0.366	0.063
Sweets	0.159	0.087	-0.117	0.424
Other Foods	0.411	0.022	0.454	0.077
Non-alcoholic Beverages	0.105	0.056	***-0*.*228***	0.011
Alcoholic Beverages	4.681	0.000	***4*.*229***	0.000
**NON-FOOD SHARES**					
	Difference	p-value	Difference	p-value
Health	-0.178	0.038	-0.200	0.120
Clothing	-0.386	0.104	-0.327	0.271
HH Costs	-7.221	0.001	-3.157	0.132
HH Energy	0.270	0.143	-0.089	0.748
Furnish and Appliances	-0.605	0.000	-0.262	0.308
Cleaning and Domestics	0.123	0.203	0.263	0.054
Transport	-1.623	0.000	***-0*.*982***	0.015
Communications	-0.768	0.000	***-0*.*583***	0.002
Gambling	0.051	0.073	-0.004	0.933
Recreation	-0.008	0.938	-0.123	0.441
Education	-0.248	0.000	-0.071	0.476
Restaurant/Hotel	0.669	0.004	-0.054	0.891
Miscellaneous	-1.006	0.000	-0.122	0.723

t-tests of conditional mean differences for tobacco consuming vs. non-tobacco consuming households for Quartile 1. The left two columns compare means for the unmatched sample and the right two columns do so for the matched sample. The column marked “Difference” is defined as the share for smoking households less the share for non-smoking households. All shares are household expenditure shares net of tobacco expenditure. The top panel reports for the food category, while the bottom panel reports for the non-food category. Statistically significant share differences (p-value < 0.05) for the matched sample are italicized and highlighted in bold.

**Table 4:** Food and non-food expenditure share differences for quartile 2.

**FOOD SHARES**					
	Difference	p-value	Difference	p-value
Grains	-1.084	0.000	-0.030	0.922
Meats	1.338	0.000	0.911	0.891
Dairy	-0.031	0.720	-0.106	0.410
Nuts and Oils	-0.234	0.000	***-0*.*233***	0.012
Fruits	-0.076	0.006	-0.026	0.536
Vegetables	-0.117	0.258	0.131	0.374
Sweets	0.167	0.041	***0*.*270***	0.026
Other Foods	0.416	0.056	0.129	0.683
Non-alcoholic Beverages	0.175	0.001	0.038	0.615
Alcoholic Beverages	2.976	0.000	***2*.*965***	0.000
**NON-FOOD SHARES**					
	Difference	p-value	Difference	p-value
Health	0.097	0.276	-0.040	0.770
Clothing	-0.050	0.828	-0.301	0.269
HH Costs	7.865	0.384	7.302	0.497
HH Energy	0.229	0.118	-0.162	0.460
Furnish and Appliances	-0.163	0.461	0.029	0.929
Cleaning and Domestics	-0.139	0.080	-0.137	0.240
Transport	-1.792	0.000	***-1*.*135***	0.028
Communications	-0.152	0.182	***-0*.*462***	0.005
Gambling	0.044	0.184	0.036	0.411
Recreation	0.072	0.518	-0.010	0.953
Education	-0.323	0.000	0.052	0.698
Restaurant/Hotel	0.271	0.153	-0.076	0.803
Miscellaneous	-0.827	0.002	0.163	0.681

t-tests of conditional mean differences for tobacco consuming vs. non-tobacco consuming households for Quartile 2. The left two columns compare means for the unmatched sample and the right two columns do so for the matched sample. The column marked “Difference” is defined as the share for smoking households less the share for non-smoking households. All shares are household expenditure shares net of tobacco expenditure. The top panel reports for the food category, while the bottom panel reports for the non-food category. Statistically significant share differences (p-value < 0.05) for the matched sample are italicized and highlighted in bold.

**Table 5:** Food and non-food expenditure share differences for quartile 3.

**FOOD SHARES**					
	Difference	p-value	Difference	p-value
Grains	-0.701	0.000	-0.090	0.694
Meats	1.215	0.000	0.503	0.834
Dairy	0.111	0.092	-0.177	0.065
Nuts and Oils	-0.130	0.001	-0.092	0.093
Fruits	-0.006	0.829	-0.013	0.725
Vegetables	0.002	0.975	0.022	0.840
Sweets	0.165	0.003	0.137	0.075
Other Foods	-0.005	0.974	-0.027	0.912
Non-alcoholic Beverages	0.077	0.065	0.068	0.299
Alcoholic Beverages	2.519	0.000	***2*.*355***	0.000
**NON-FOOD SHARES**					
	Difference	p-Value	Difference	p-Value
Health	0.308	0.000	0.200	0.074
Clothing	0.358	0.079	-0.183	0.330
HH Costs	-0.726	0.833	0.172	0.953
HH Energy	0.179	0.120	***-0*.*558***	0.002
Furnish and Appliances	0.117	0.590	0.454	0.137
Cleaning and Domestics	-0.071	0.399	0.083	0.486
Transport	-0.869	0.024	-0.429	0.448
Communications	0.014	0.883	-0.182	0.229
Gambling	0.056	0.006	***0*.*079***	0.005
Recreation	0.376	0.000	***0*.*423***	0.005
Education	-0.951	0.000	***-0*.*601***	0.008
Restaurant/Hotel	0.349	0.032	***0*.*574***	0.008
Miscellaneous	-0.986	0.003	0.189	0.682

t-tests of conditional mean differences for tobacco consuming vs. non-tobacco consuming households for Quartile 3. The left two columns compare means for the unmatched sample and the right two columns do so for the matched sample. The column marked “Difference” is defined as the share for smoking households less the share for non-smoking households. All shares are household expenditure shares net of tobacco expenditure. The top panel reports for the food category, while the bottom panel reports for the non-food category. Statistically significant share differences (p-value < 0.05) for the matched sample are italicized and highlighted in bold.

**Table 6:** Food and non-food expenditure share differences for quartile 3.

**FOOD SHARES**					
	Difference	p-value	Difference	p-value
Grains	0.084	0.278	0.010	0.910
Meats	0.941	0.000	0.361	0.837
Dairy	0.203	0.000	0.041	0.427
Nuts and Oils	0.009	0.611	0.001	0.968
Fruits	-0.012	0.432	-0.032	0.131
Vegetables	0.200	0.000	0.043	0.407
Sweets	0.135	0.000	0.027	0.463
Other Foods	-0.126	0.274	0.045	0.773
Non-alcoholic Beverages	0.098	0.000	0.031	0.376
Alcoholic Beverages	1.145	0.000	***1*.*169***	0.000
**NON-FOOD SHARES**					
	Difference	p-value	Difference	p-value
Health	0.093	0.258	0.070	0.549
Clothing	0.039	0.786	0.244	0.797
HH Costs	0.332	0.691	-1.650	0.101
HH Energy	0.305	0.001	0.031	0.822
Furnish and Appliances	-0.270	0.044	0.221	0.210
Cleaning and Domestics	0.029	0.805	0.024	0.881
Transport	-0.981	0.022	0.157	0.785
Communications	0.105	0.212	***-0*.*258***	0.022
Gambling	0.084	0.004	***0*.*093***	0.014
Recreation	0.357	0.001	0.165	0.248
Education	-0.505	0.006	-0.296	0.235
Restaurant/Hotel	0.514	0.000	***0*.*394***	0.044
Miscellaneous	-1.106	0.011	-0.402	0.492

t-tests of conditional mean differences for tobacco consuming vs. non-tobacco consuming households for Quartile 4. The left two columns compare means for the unmatched sample and the right two columns do so for the matched sample. The column marked “Difference” is defined as the share for smoking households less the share for non-smoking households. All shares are household expenditure shares net of tobacco expenditure. The top panel reports for the food category, while the bottom panel reports for the non-food category. Statistically significant share differences (p-value < 0.05) for the matched sample are italicized and highlighted in bold.

#### Results for quartile 1

The first quartile of the expenditure distribution (net of tobacco expenditure) with monthly expenditure ranging between ZAR18.25 (USD2.70) and ZAR1,739.08 (USD256.88), using a March 2011 exchange rate of ZAR6.77 to USD1, covers the poorest households. The highest amount of monthly expenditure on tobacco in this quartile is ZAR819.00 (USD120.97), although the average expenditure is only ZAR18.27 (USD2.70). The tobacco budget share among tobacco consuming households is 6.78%. This is the highest tobacco budget share among the four quartiles showing that tobacco expenditure tends to be regressive.

Expenditure share differentials between unmatched and matched households in quartile one are presented in Table 3. As can be seen, before matching there are a total of 12 expenditure items (across food and non-food categories) for which smoking households allocate smaller shares that are statistically significant (at the 5% level). After matching, this reduces to only 3 items under food (dairy, fruits and non-alcoholic beverages) and 2 items under non-food (transport and communications). Similarly for crowding-in and before matching, there are 4 items across food and non-food where expenditure shares for smoking households are bigger than non-smoking households. This reduces to only alcoholic beverages after matching.

#### Results for quartile 2

The results for quartile 2 are reported in Table 4. This quartile contains households with monthly expenditure ranging from ZAR1,758.83 (USD256.93) to ZAR3,136.00 (USD463.22). The maximum monthly tobacco expenditure in this quartile is ZAR1,141.58 (USD168.62) and the average household spends ZAR18.76 (USD2.77) on tobacco per month. The share of the household budget dedicated to tobacco is only 2.78%, slightly less than half of the Quartile one share.

In Table 4, there are a total of six expenditure items across food and non-food where crowding out is observed (at the 5% level of significance) before the matching is done. After matching, this reduces to only one item for food (nuts and oils) and two items under the non-food category (transport and communications). Pre-match crowding-in is only observed in the food category for four items (meats, sweets, non-alcoholic and alcoholic beverages). After matching, only sweets and alcoholic beverages show up as instances where smoking households allocate greater shares than non-smoking households at the 5% level of significance.

#### Results for quartile 3

The results for the third quartile are contained in Table 5. This quartile contains households with monthly expenditures ranging from ZAR3,136.08 (USD463.22) to ZAR6,763.42 (USD999.02). The highest monthly tobacco expenditure is ZAR1,078.08 (USD159.24) and the average is ZAR35.79 (USD5.29). The average budget share allocation among consuming households is 3.52%.

According to Table 5, crowding-out prior to matching (at the 5% level) is observed for 5 expenditure items across food and non-food. After the matching is done, only two expenditure items (education and household energy) are crowded-out, and interestingly all of them are part of the non-food category. In as far as crowding-in is concerned, 7 items are observed as having been crowded-in before matching– 3 under the food category and 4 under the non-food category. After matching, only 4 items remain as being crowded-in: alcoholic beverages under the food category and the rest under the non-food category (gambling, recreation and restaurant/hotel).

#### Results for quartile 4

The results for the forth quartile are contained in Table 6. In quartile 4, monthly expenditure ranges from ZAR6,764.33 (USD999.16) to ZAR312,264.84 (USD46,124.79). The highest amount spent on tobacco per month in this quartile is ZAR3,597.00 (USD531.31) and the monthly average spend on tobacco is ZAR72.40 (USD10.69). The average tobacco budget share, among consuming households, is 2.17%.

In Table 6, we see that only four items, and all in the non-food category, are crowded-out prior to matching at the 5% level of significance. After matching, only communications expenditure shows up as being crowded-out. In terms of crowding-in, 9 items between the food and non-food categories show up crowded-in in the table. This reduces to only 3 items (alcoholic beverages, gambling and restaurant/hotel) when comparing matched households.

## Discussion

This section of the paper discusses the results presented in Tables 3–6. The first thing to notice about the results is that the number of expenditure items that are crowded-in/crowded-out is always greater when comparing unmatched households than when comparing matched households. This is because, as evidenced in Table 1, smoking households and non-smoking households are rather different in many important respects (especially in household structure) and this difference influences expenditure allocations over and above whatever influence tobacco expenditure might have. Analysts, therefore, have to be careful before making any direct comparisons between the two types of households.

Second, a consistent finding across all four quartiles is that smoking households have a bigger expenditure share allocation towards alcoholic beverages than non-smoking households. The shares differentials range between 4 percentage points for the poorest quartile to 1 percentage point for the richest quartile. This crowding-in effect on alcohol is one of the most consistent findings in the literature. A wide range of studies using different datasets and techniques and across different countries often find that spending on tobacco does lead households to spend more on alcohol. In other words, tobacco and alcohol are complementary goods [20].

Another result of note from Tables 3–6 is that food items are only crowded-out in the bottom two quartiles of the net expenditure distribution. Dairy, fruits and non-alcoholic beverages are crowded out in quartile 1 with expenditure share differences of 0.8, 0.1 and 0.2 percentage points respectively when compared to non-smoking households. Nuts and oils are crowded out in quartile 2 with smoking households allocating 0.2 percentage points lower than non-smoking households. The finding that food is more likely to be crowded-out for poorer households than well-off households has also been found in India, Indonesia, South Africa and Zambia [4, 6, 8, 21]. These finding suggest that poorer smoking households face budget constraints that are more binding than those faced by well-off households resulting in the sacrifice of some food items. Expenditure on transport and communications is also consistently crowded-out for the bottom 2 quartiles in Tables 3–6. For transport, smoking households’ allocation is a percentage point smaller than non-smoking households while for communication, the magnitude is about half of what it is for transport expenditure. Out-of-pocket expenditures on especially transportation are important for poor households in South Africa given the absence of a properly functioning public transportation system [22]. An earlier study on South Africa also found that transport expenditure was crowded out by household expenditure on tobacco [6].

Interestingly, the results in Tables 3–6 show that some “luxury” items are consistently crowded-in by tobacco expenditure but only for well-off households. Smoking households in the top 2 quartiles have larger expenditure shares allocated to gambling and restaurant/hotel expenditures. This is also not surprising given that expenditure on luxury items is often complementary to expenditure on tobacco especially for households whose budget constraints are not as binding as they are for poorer households.

## Conclusion

In this paper we took seriously the challenge of attributing causation in the literature on the crowding-out effect of tobacco expenditure. As argued earlier, the endogeneity of tobacco expenditure in systems of demand equations is rarely confronted, and, when confronted, it is done using less than satisfactory instrumental variables. The instrumental variable of choice in the literature has been the adult sex ratio given that adult males are often more likely to smoke than adult females [4]. Chelwa and Van Walbeek (2014) have argued that the adult sex ratio is unlikely to meet the exclusion restriction required of instrumental variables, given that it is just as likely to influence household expenditure patterns as it is to influence tobacco expenditure [8]. Imposing some assumptions on the direction of correlation between the adult sex ratio and the error term can help overcome this problem [8]. However, the assumptions imposed often appear ad hoc and, by definition, rule out the possibility of crowding-in.

In this paper we use a more transparent and data-driven approach—genetic matching—to deal with endogeneity concerns. Genetic matching, a general version of propensity score matching, does not require ad hoc assumptions about the validity of an instrumental variable. And, unlike propensity score matching, genetic matching transparently provides measures of balance giving the practitioner a more objective assessment of the success of the matching algorithm. Our analysis confirms some of the findings in the literature. In particular, we find that the crowding-out of food is a low-income phenomenon in South Africa, a finding in line with previous South African research and in line with studies elsewhere [4, 6–8]. Much like previous work, we find that tobacco households consistently allocate a bigger expenditure share to alcohol than non-tobacco consuming households. That is, we find that tobacco and alcohol are complements.

## Supporting information

S1 Fig Densities of household income and expenditure after one-to-one genetic matching for Quartile 1. Panel (a) illustrates the density for the natural log of household income after matching. Panel (b) does the same for the natural log of household expenditure net of tobacco purchases.
(PDF)

S2 Fig Densities of household income and expenditure after one-to-one genetic matching for Quartile 2. Panel (a) illustrates the density for the natural log of household income after matching. Panel (b) does the same for the natural log of household expenditure net of tobacco purchases.
(PDF)

S3 Fig Densities of household income and expenditure after one-to-one genetic matching for Quartile 3. Panel (a) illustrates the density for the natural log of household income after matching. Panel (b) does the same for the natural log of household expenditure net of tobacco purchases.
(PDF)

S4 Fig Densities of household income and expenditure after one-to-one genetic matching for Quartile 4. Panel (a) illustrates the density for the natural log of household income after matching. Panel (b) does the same for the natural log of household expenditure net of tobacco purchases.
(PDF)

S1 Table Descriptive statistics before matching for Quartile 1 2010. (DOCX)

S2 Table Descriptive statistics after matching for Quartile 1 2010. (DOCX)

S3 Table Descriptive statistics before matching for Quartile 2 2010. (DOCX)

S4 Table Descriptive statistics after matching for Quartile 2 2010. (DOCX)

S5 Table Descriptive statistics before matching for Quartile 3 2010. (DOCX)

S6 Table Descriptive statistics after matching for Quartile 3 2010. (DOCX)

S7 Table Descriptive statistics before matching for Quartile 4 2010. (DOCX)

S8 Table Descriptive statistics after matching for Quartile 4 2010. (DOCX)
