# Supplementary figures and images for: The effect of tobacco expenditure on expenditure shares in South African households: A genetic matching approach
Source: PLoS One. 2019 Sep 6;14(9):e0222000. doi: 10.1371/journal.pone.0222000 (PMC6730990; doi:10.1371/journal.pone.0222000)

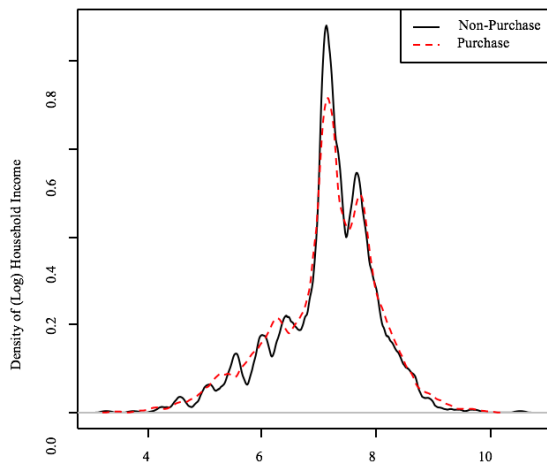

(a) Log HH Income: Quartile 1

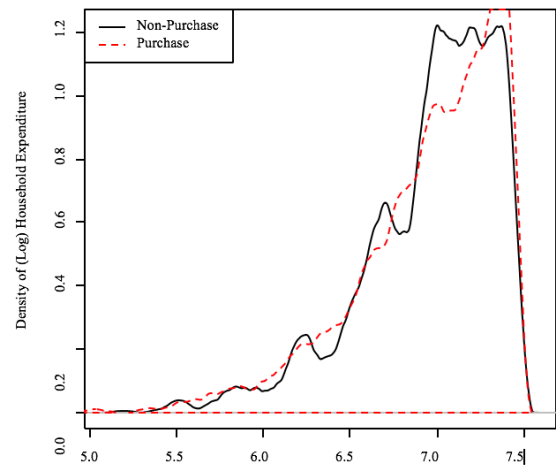

(b) Log HH Expenditure: Quartile 1

Supplement: S1 Fig — Panel (a) illustrates the density for the natural log of household income after matching. Panel (b) does the same for the natural log of household expenditure net of tobacco purchases. (PDF) [file pone.0222000.s001.pdf]

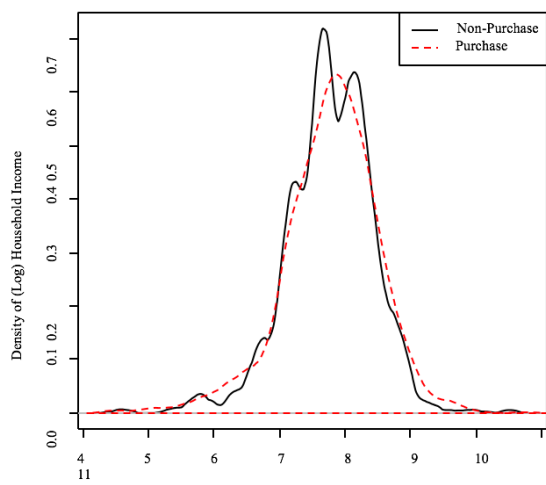

(a) Log HH Income: Quartile 2 2010

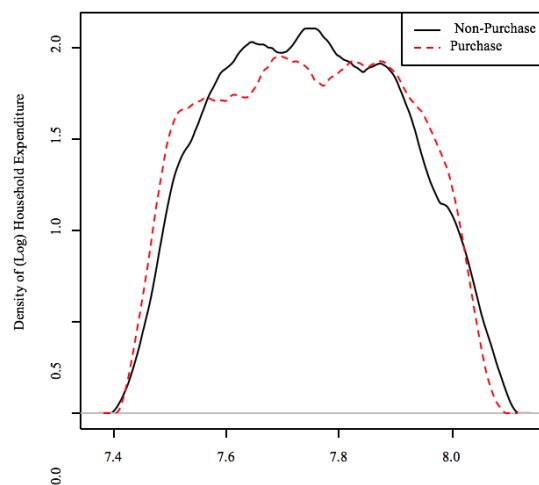

(b) Log HH Expenditure: Quartile 2

Supplement: S2 Fig — Panel (a) illustrates the density for the natural log of household income after matching. Panel (b) does the same for the natural log of household expenditure net of tobacco purchases. (PDF) [file pone.0222000.s002.pdf]

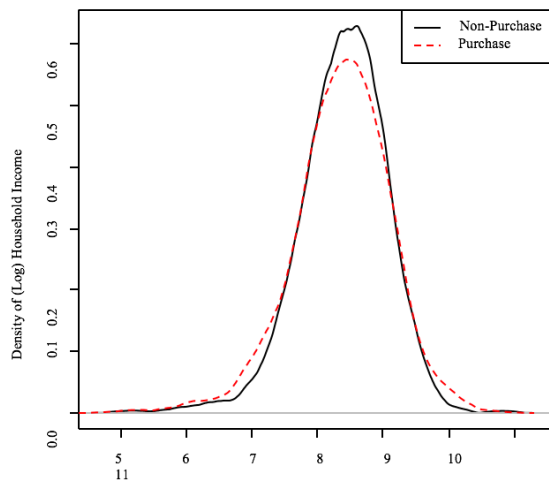

(a) Log HH Income: Quartile 3

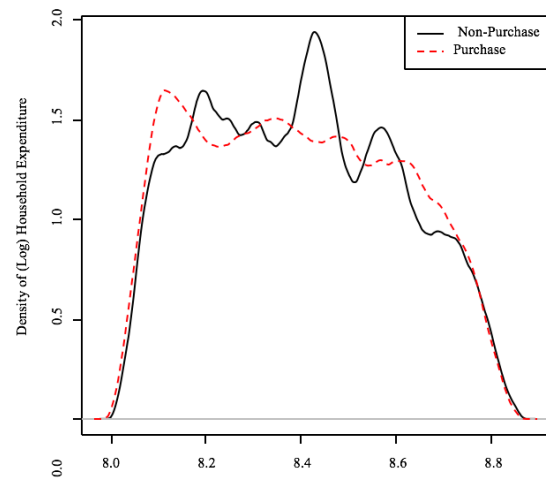

(b) Log HH Expenditure: Quartile 3

Supplement: S3 Fig — Panel (a) illustrates the density for the natural log of household income after matching. Panel (b) does the same for the natural log of household expenditure net of tobacco purchases. (PDF) [file pone.0222000.s003.pdf]

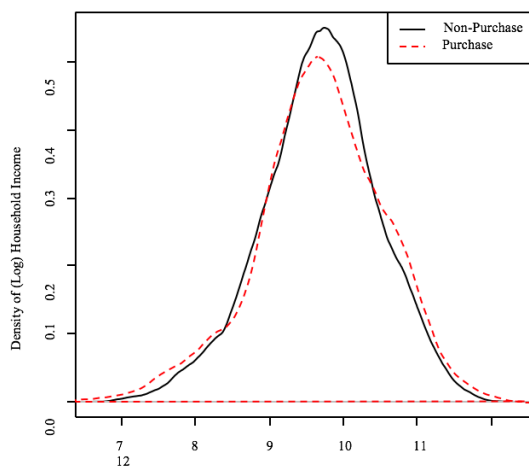

(a) Log HH Income: Quartile 4

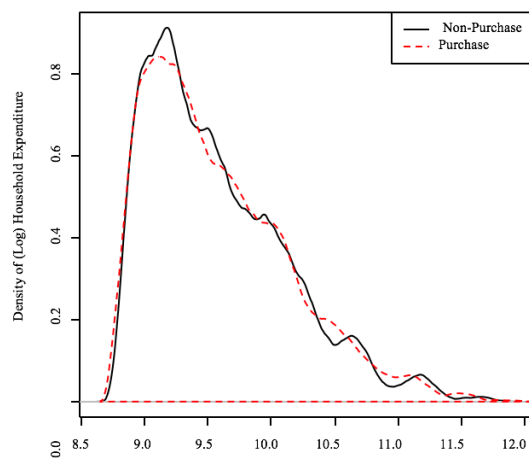

(b) Log HH Expenditure: Quartile 4

Supplement: S4 Fig — Panel (a) illustrates the density for the natural log of household income after matching. Panel (b) does the same for the natural log of household expenditure net of tobacco purchases. (PDF) [file pone.0222000.s004.pdf]
