# Supplementary material for: The effect of tobacco expenditure on expenditure shares in South African households: A genetic matching approach
Source: PLoS One. 2019 Sep 6;14(9):e0222000. doi: 10.1371/journal.pone.0222000 (PMC6730990; doi:10.1371/journal.pone.0222000)
Supplement: S1 Table — (DOCX) [file pone.0222000.s005.docx]

**S1 Table. Descriptive statistics before matching for Quartile 1 2010.**

| **Variable** | **Non-Smoking Average** | **Smoking Average** | **t-probability** | **ks-probability** |
| --- | --- | --- | --- | --- |
| Propensity Score | 0.225 | 0.371 | 0 | 0 |
| HH Head Age Group | 9.819 | 9.959 | 0.136 | 0.002 |
| HH Head Schooling | 1.369 | 1.272 | 0 | 0 |
| HH Head Training | 0.085 | 0.053 | 0 |  |
| Black HH Head | 0.963 | 0.854 | 0 |  |
| Coloured HH Head | 0.035 | 0.144 | 0 |  |
| White HH Head | 0.002 | 0.002 | 0.915 |  |
| Female HH Head | 0.444 | 0.74 | 0 |  |
| Black HH Log Inc | 6.838 | 6.043 | 0 | 0 |
| Coloured HH Log Inc | 0.265 | 1.065 | 0 | 0 |
| White HH Log Inc | 0.015 | 0.015 | 0.994 | 0.834 |
| Female Head Log Inc | 3.183 | 5.261 | 0 | 0 |
| Log Net Exp | 6.957 | 6.951 | 0.612 | 0.04 |
| Black HH Log Net Exp | 6.694 | 5.921 | 0 | 0 |
| Coloured HH Log Net Exp | 0.249 | 1.017 | 0 | 0 |
| White HH Log Net Exp | 0.014 | 0.013 | 0.924 | 0.942 |
| Female Head Log Net Exp | 3.071 | 5.127 | 0 | 0 |
| Black HH Sex Ratio | 0.416 | 0.586 | 0 | 0 |
| Coloured HH Sex Ratio | 0.015 | 0.078 | 0 | 0 |
| White HH Sex Ratio | 0.001 | 0.001 | 0.901 | 0.788 |
| Female Head Sex Ratio | 0.334 | 0.59 | 0 | 0 |
| Black HH Adult Ratio | 0.754 | 0.755 | 0.918 | 0 |
| Coloured HH Adult Ratio | 0.028 | 0.119 | 0 | 0 |
| White HH Adult Ratio | 0.002 | 0.002 | 0.767 | 0.775 |
| Female Head Adult Ratio | 0.382 | 0.672 | 0 | 0 |
| Girls (0-4) in HH | 0.166 | 0.115 | 0 | 0 |
| Boys (0-4) in HH | 0.174 | 0.093 | 0 | 0 |
| Girls (5-14) in HH | 0.326 | 0.184 | 0 | 0 |
| Boys (5-14) in HH | 0.311 | 0.178 | 0 | 0 |
| Women (15-64) in HH | 0.979 | 0.636 | 0 | 0 |
| Men (15-64) in HH | 0.794 | 1.034 | 0 | 0 |
| Women (65+) in HH | 0.191 | 0.135 | 0 | 0 |
| Men (65+) in HH | 0.081 | 0.135 | 0 | 0 |
| Eastern Cape | 0.034 | 0.088 | 0 |  |
| Western Cape | 0.168 | 0.151 | 0.116 |  |
| Northern Cape | 0.034 | 0.105 | 0 |  |
| Free State | 0.068 | 0.159 | 0 |  |
| Kwa-Zulu Natal | 0.179 | 0.096 | 0 |  |
| Northwest Province | 0.111 | 0.138 | 0.007 |  |
| Gauteng Province | 0.109 | 0.092 | 0.055 |  |
| Mpumulanga Province | 0.098 | 0.078 | 0.012 |  |
| Urban | 0.462 | 0.564 | 0 |  |
| Obs. |  | 4517 |  | 1615 |
