# Supplementary material for: The effect of tobacco expenditure on expenditure shares in South African households: A genetic matching approach
Source: PLoS One. 2019 Sep 6;14(9):e0222000. doi: 10.1371/journal.pone.0222000 (PMC6730990; doi:10.1371/journal.pone.0222000)
Supplement: S2 Table — (DOCX) [file pone.0222000.s006.docx]

**S2 Table. Descriptive statistics after matching for Quartile 1 2010.**

| **Variable name** | **Smoking Average** | **Non-smoking Average** | **t-probability** | **ks-probability** |
| --- | --- | --- | --- | --- |
| Propensity Score | 0.37 | 0.371 | 0.098 | 0.98 |
| HH Head Age Group | 10.067 | 9.959 | 0.187 | 0.142 |
| HH Head Schooling | 1.266 | 1.272 | 0.44 | 0.18 |
| HH Head Training | 0.041 | 0.053 | 0.002 |  |
| Black HH Head | 0.854 | 0.854 | 1 |  |
| Coloured HH Head | 0.144 | 0.144 | 1 |  |
| White HH Head | 0.002 | 0.002 | 1 |  |
| Female HH Head | 0.741 | 0.74 | 0.317 |  |
| Black HH Log Inc | 6.049 | 6.043 | 0.234 | 0.339 |
| Coloured HH Log Inc | 1.07 | 1.065 | 0.514 | 0.57 |
| White HH Log Inc | 0.014 | 0.014 | 0.118 | 0.991 |
| Female Head Log Inc | 5.276 | 5.261 | 0.103 | 0.409 |
| Black HH Log Net Exp | 6.96 | 6.951 | 0.204 | 0.113 |
| Coloured HH Log Net Exp | 5.929 | 5.921 | 0.219 | 0.192 |
| White HH Log Net Exp | 1.018 | 1.017 | 0.697 | 0.482 |
| Female Head Log Net Exp | 0.013 | 0.013 | 0.191 | 0.991 |
| Log Net Exp | 5.138 | 5.127 | 0.151 | 0.1 |
| Black HH Sex Ratio | 0.587 | 0.586 | 0.111 | 1 |
| Coloured HH Sex Ratio | 0.078 | 0.078 | 0.22 | 0.979 |
| White HH Sex Ratio | 0.001 | 0.001 | 1 | 1 |
| Female Head Sex Ratio | 0.591 | 0.59 | 0.392 | 0.997 |
| Black HH Adult Ratio | 0.754 | 0.755 | 0.396 | 0.964 |
| Coloured HH Adult Ratio | 0.118 | 0.119 | 0.394 | 0.881 |
| White HH Adult Ratio | 0.002 | 0.002 | 1 | 1 |
| Female Head Adult Ratio | 0.671 | 0.672 | 0.589 | 0.999 |
| Girls (0-4) in HH | 0.105 | 0.115 | 0.31 | 0.51 |
| Boys (0-4) in HH | 0.105 | 0.093 | 0.25 | 0.087 |
| Girls (5-14) in HH | 0.174 | 0.185 | 0.383 | 0.38 |
| Boys (5-14) in HH | 0.167 | 0.178 | 0.372 | 0.688 |
| Women (15-64) in HH | 0.602 | 0.636 | 0.013 | 0.258 |
| Men (15-64) in HH | 1.012 | 1.034 | 0.227 | 0.506 |
| Women (65+) in HH | 0.152 | 0.135 | 0.059 | 0.136 |
| Men (65+) in HH | 0.168 | 0.135 | 0.002 | 0.006 |
| Eastern Cape | 0.098 | 0.089 | 0.265 |  |
| Western Cape | 0.155 | 0.151 | 0.74 |  |
| Northern Cape | 0.086 | 0.105 | 0.027 |  |
| Free State | 0.154 | 0.159 | 0.591 |  |
| Kwa-Zulu Natal | 0.097 | 0.096 | 0.945 |  |
| Northwest Province | 0.124 | 0.137 | 0.199 |  |
| Gauteng Province | 0.098 | 0.092 | 0.545 |  |
| Mpumulanga Province | 0.09 | 0.078 | 0.199 |  |
| Urban | 0.545 | 0.564 | 0.221 |  |
| Observations | 1615 | 1615 |  |  |
