# Supplementary material for: The effect of tobacco expenditure on expenditure shares in South African households: A genetic matching approach
Source: PLoS One. 2019 Sep 6;14(9):e0222000. doi: 10.1371/journal.pone.0222000 (PMC6730990; doi:10.1371/journal.pone.0222000)
Supplement: S3 Table — (DOCX) [file pone.0222000.s007.docx]

**S 3 Table. Descriptive statistics before matching for Quartile 2 2010.**

| **Variable name** | **smoking average** | **non-smoking average** | **t-probability** | **ks-probability** |
| --- | --- | --- | --- | --- |
| Propensity Score | 0.196 | 0.294 | 0 | 0 |
| HH Head Age Group | 10.262 | 10.225 | 0.708 | 0.102 |
| HH Head Schooling | 1.495 | 1.377 | 0 | 0 |
| HH Head Training | 0.123 | 0.084 | 0 |  |
| Black HH Head | 0.946 | 0.805 | 0 |  |
| Coloured HH Head | 0.051 | 0.188 | 0 |  |
| White HH Head | 0.004 | 0.007 | 0.181 |  |
| Female HH Head | 0.447 | 0.63 | 0 |  |
| Black HH Log Inc | 7.28 | 6.237 | 0 | 0 |
| Coloured HH Log Inc | 0.396 | 1.486 | 0 | 0 |
| White HH Log Inc | 0.029 | 0.054 | 0.197 | 0.17 |
| Female Head Log Inc | 3.502 | 4.927 | 0 | 0 |
| Log Net Exp | 7.759 | 7.744 | 0.002 | 0.001 |
| Black HH Log Net Exp | 7.338 | 6.232 | 0 | 0 |
| Coloured HH Log Net Exp | 0.393 | 1.459 | 0 | 0 |
| White HH Log Net Exp | 0.028 | 0.053 | 0.188 | 0.175 |
| Female Head Log Net Exp | 3.471 | 4.88 | 0 | 0 |
| Black HH Sex Ratio | 0.395 | 0.46 | 0 | 0 |
| Coloured HH Sex Ratio | 0.02 | 0.087 | 0 | 0 |
| White HH Sex Ratio | 0.001 | 0.003 | 0.12 | 0.026 |
| Female Head Sex Ratio | 0.298 | 0.431 | 0 | 0 |
| Black HH Adult Ratio | 0.69 | 0.646 | 0 | 0 |
| Coloured HH Adult Ratio | 0.037 | 0.145 | 0 | 0 |
| White HH Adult Ratio | 0.004 | 0.005 | 0.4 | 0.154 |
| Female Head Adult Ratio | 0.356 | 0.524 | 0 | 0 |
| Girls (0-4) in HH | 0.256 | 0.188 | 0 | 0 |
| Boys (0-4) in HH | 0.238 | 0.201 | 0.014 | 0.02 |
| Girls (5-14) in HH | 0.444 | 0.327 | 0 | 0 |
| Boys (5-14) in HH | 0.448 | 0.358 | 0 | 0 |
| Women (15-64) in HH | 1.256 | 1.049 | 0 | 0 |
| Men (15-64) in HH | 0.97 | 1.24 | 0 | 0 |
| Women (65+) in HH | 0.232 | 0.21 | 0.113 | 0.076 |
| Men (65+) in HH | 0.104 | 0.136 | 0.004 | 0.004 |
| Eastern Cape | 0.052 | 0.159 | 0 |  |
| Western Cape | 0.154 | 0.123 | 0.003 |  |
| Northern Cape | 0.038 | 0.068 | 0 |  |
| Free State | 0.069 | 0.162 | 0 |  |
| Kwa-Zulu Natal | 0.174 | 0.087 | 0 |  |
| Northwest Province | 0.106 | 0.105 | 0.898 |  |
| Gauteng Province | 0.127 | 0.112 | 0.13 |  |
| Mpumulanga Province | 0.109 | 0.096 | 0.166 |  |
| Urban | 0.513 | 0.633 | 0 |  |
| Observations | 4727 | 1312 |  |  |
