# Supplementary material for: The effect of tobacco expenditure on expenditure shares in South African households: A genetic matching approach
Source: PLoS One. 2019 Sep 6;14(9):e0222000. doi: 10.1371/journal.pone.0222000 (PMC6730990; doi:10.1371/journal.pone.0222000)
Supplement: S4 Table — (DOCX) [file pone.0222000.s008.docx]

**S 4 Table. Descriptive statistics after matching for Quartile 2 2010.**

| **Variable name** | **Smoking average** | **Non-smoking average** | **t-probability** | **ks-probability** |
| --- | --- | --- | --- | --- |
| Propensity Score | 0.293 | 0.294 | 0.174 | 0.996 |
| HH Head Age Group | 10.345 | 10.225 | 0.211 | 0.813 |
| HH Head Schooling | 1.39 | 1.377 | 0.18 | 0.548 |
| HH Head Training | 0.074 | 0.084 | 0.047 |  |
| Black HH Head | 0.805 | 0.805 | 1 |  |
| Coloured HH Head | 0.188 | 0.188 | 1 |  |
| White HH Head | 0.007 | 0.007 | 1 |  |
| Female HH Head | 0.63 | 0.63 | 1 |  |
| Black HH Log Inc | 6.238 | 6.237 | 0.872 | 0.818 |
| Coloured HH Log Inc | 1.474 | 1.486 | 0.179 | 0.128 |
| White HH Log Inc | 0.054 | 0.054 | 0.952 | 0.852 |
| Female Head Log Inc | 4.93 | 4.927 | 0.78 | 0.686 |
| Log Net Exp | 7.749 | 7.744 | 0.176 | 0.18 |
| Black HH Log Net Exp | 6.233 | 6.232 | 0.591 | 0.635 |
| Coloured HH Log Net Exp | 1.462 | 1.459 | 0.221 | 0.674 |
| White HH Log Net Exp | 0.054 | 0.053 | 0.225 | 0.879 |
| Female Head Log Net Exp | 4.883 | 4.88 | 0.295 | 0.408 |
| Black HH Sex Ratio | 0.46 | 0.46 | 0.612 | 0.841 |
| Coloured HH Sex Ratio | 0.087 | 0.087 | 0.178 | 0.757 |
| White HH Sex Ratio | 0.003 | 0.003 | 0.786 | 0.997 |
| Female Head Sex Ratio | 0.431 | 0.431 | 0.961 | 0.996 |
| Black HH Adult Ratio | 0.646 | 0.646 | 0.637 | 0.903 |
| Coloured HH Adult Ratio | 0.146 | 0.145 | 0.625 | 0.975 |
| White HH Adult Ratio | 0.007 | 0.005 | 0.034 | 0.385 |
| Female Head Adult Ratio | 0.526 | 0.524 | 0.229 | 0.991 |
| Girls (0-4) in HH | 0.182 | 0.188 | 0.696 | 0.981 |
| Boys (0-4) in HH | 0.188 | 0.201 | 0.412 | 0.669 |
| Girls (5-14) in HH | 0.306 | 0.327 | 0.258 | 0.24 |
| Boys (5-14) in HH | 0.319 | 0.358 | 0.054 | 0.496 |
| Women (15-64) in HH | 1.005 | 1.049 | 0.057 | 0.658 |
| Men (15-64) in HH | 1.182 | 1.24 | 0.014 | 0.017 |
| Women (65+) in HH | 0.219 | 0.21 | 0.539 | 0.355 |
| Men (65+) in HH | 0.152 | 0.136 | 0.197 | 0.258 |
| Eastern Cape | 0.181 | 0.159 | 0.009 |  |
| Western Cape | 0.123 | 0.123 | 0.949 |  |
| Northern Cape | 0.083 | 0.068 | 0.05 |  |
| Free State | 0.123 | 0.162 | 0 |  |
| Kwa-Zulu Natal | 0.078 | 0.087 | 0.296 |  |
| Northwest Province | 0.097 | 0.105 | 0.429 |  |
| Gauteng Province | 0.12 | 0.112 | 0.436 |  |
| Mpumulanga Province | 0.099 | 0.096 | 0.771 |  |
| Urban | 0.652 | 0.633 | 0.248 |  |
| Observations | 1312 | 1312 |  |  |
