# Supplementary material for: The effect of tobacco expenditure on expenditure shares in South African households: A genetic matching approach
Source: PLoS One. 2019 Sep 6;14(9):e0222000. doi: 10.1371/journal.pone.0222000 (PMC6730990; doi:10.1371/journal.pone.0222000)
Supplement: S5 Table — (DOCX) [file pone.0222000.s009.docx]

**S 5 Table. Descriptive statistics before matching for Quartile 3**

| **Variable name** | **Smoking average** | **Non-smoking average** | **t-probability** | **ks-probability** |
| --- | --- | --- | --- | --- |
| Propensity Score | 0.207 | 0.302 | 0 | 0 |
| HH Head Age Group | 10.452 | 10.536 | 0.37 | 0.021 |
| HH Head Schooling | 1.776 | 1.623 | 0 | 0 |
| HH Head Training | 0.174 | 0.129 | 0 |  |
| Black HH Head | 0.896 | 0.689 | 0 |  |
| Coloured HH Head | 0.08 | 0.279 | 0 |  |
| White HH Head | 0.023 | 0.032 | 0.084 |  |
| Female HH Head | 0.498 | 0.642 | 0 |  |
| Black HH Log Inc | 7.411 | 5.707 | 0 | 0 |
| Coloured HH Log Inc | 0.688 | 2.379 | 0 | 0 |
| White HH Log Inc | 0.198 | 0.279 | 0.075 | 0.035 |
| Female Head Log Inc | 4.19 | 5.388 | 0 | 0 |
| Log Net Exp | 8.395 | 8.392 | 0.678 | 0.187 |
| Black HH Log Net Exp | 7.519 | 5.776 | 0 | 0 |
| Coloured HH Log Net Exp | 0.678 | 2.341 | 0 | 0 |
| White HH Log Net Exp | 0.198 | 0.275 | 0.084 | 0.081 |
| Female Head Log Net Exp | 4.188 | 5.392 | 0 | 0 |
| Black HH Sex Ratio | 0.379 | 0.381 | 0.852 | 0 |
| Coloured HH Sex Ratio | 0.033 | 0.129 | 0 | 0 |
| White HH Sex Ratio | 0.008 | 0.017 | 0.002 | 0 |
| Female Head Sex Ratio | 0.302 | 0.404 | 0 | 0 |
| Black HH Adult Ratio | 0.646 | 0.536 | 0 | 0 |
| Coloured HH Adult Ratio | 0.061 | 0.212 | 0 | 0 |
| White HH Adult Ratio | 0.021 | 0.029 | 0.118 | 0.067 |
| Female Head Adult Ratio | 0.382 | 0.516 | 0 | 0 |
| Girls (0-4) in HH | 0.25 | 0.237 | 0.42 | 0.36 |
| Boys (0-4) in HH | 0.261 | 0.228 | 0.031 | 0.027 |
| Girls (5-14) in HH | 0.48 | 0.408 | 0.001 | 0.014 |
| Boys (5-14) in HH | 0.497 | 0.423 | 0.001 | 0.001 |
| Women (15-64) in HH | 1.402 | 1.315 | 0.007 | 0.015 |
| Men (15-64) in HH | 1.105 | 1.427 | 0 | 0 |
| Women (65+) in HH | 0.244 | 0.233 | 0.444 | 0.381 |
| Men (65+) in HH | 0.127 | 0.152 | 0.024 | 0.022 |
| Eastern Cape | 0.087 | 0.238 | 0 |  |
| Western Cape | 0.131 | 0.098 | 0.001 |  |
| Northern Cape | 0.042 | 0.06 | 0.008 |  |
| Free State | 0.077 | 0.146 | 0 |  |
| Kwa-Zulu Natal | 0.143 | 0.071 | 0 |  |
| Northwest Province | 0.105 | 0.103 | 0.874 |  |
| Gauteng Province | 0.166 | 0.156 | 0.363 |  |
| Mpumulanga Province | 0.102 | 0.075 | 0.001 |  |
| Urban | 0.617 | 0.764 | 0 |  |
| Observations | 4683 | 1393 |  |  |
