# Supplementary material for: The effect of tobacco expenditure on expenditure shares in South African households: A genetic matching approach
Source: PLoS One. 2019 Sep 6;14(9):e0222000. doi: 10.1371/journal.pone.0222000 (PMC6730990; doi:10.1371/journal.pone.0222000)
Supplement: S6 Table — (DOCX) [file pone.0222000.s010.docx]

**S 6 Table. Descriptive statistics after matching for Quartile 3**

| **Variable name** | **Smoking average** | **Non-smoking average** | **t-probability** | **ks-probability** |
| --- | --- | --- | --- | --- |
| Propensity Score | 0.301 | 0.302 | 0.033 | 0.447 |
| HH Head Age Group | 10.795 | 10.536 | 0.005 | 0.084 |
| HH Head Schooling | 1.622 | 1.623 | 0.763 | 0.998 |
| HH Head Training | 0.118 | 0.128 | 0.116 |  |
| Black HH Head | 0.689 | 0.689 | 1 |  |
| Coloured HH Head | 0.279 | 0.279 | 1 |  |
| White HH Head | 0.032 | 0.032 | 1 |  |
| Female HH Head | 0.642 | 0.642 | 1 |  |
| Black HH Log Inc | 5.734 | 5.707 | 0.036 | 0.431 |
| Coloured HH Log Inc | 2.384 | 2.379 | 0.519 | 0.469 |
| White HH Log Inc | 0.278 | 0.279 | 0.629 | 0.301 |
| Female Head Log Inc | 5.406 | 5.388 | 0.136 | 0.407 |
| Log Net Exp | 8.397 | 8.392 | 0.061 | 0.256 |
| Black HH Log Net Exp | 5.777 | 5.776 | 0.918 | 0.95 |
| Coloured HH Log Net Exp | 2.345 | 2.341 | 0.034 | 0.296 |
| White HH Log Net Exp | 0.276 | 0.275 | 0.457 | 0.848 |
| Female Head Log Net Exp | 5.396 | 5.392 | 0.05 | 0.493 |
| Black HH Sex Ratio | 0.38 | 0.381 | 0.406 | 0.818 |
| Coloured HH Sex Ratio | 0.128 | 0.129 | 0.696 | 0.371 |
| White HH Sex Ratio | 0.017 | 0.017 | 0.955 | 0.799 |
| Female Head Sex Ratio | 0.403 | 0.404 | 0.404 | 0.347 |
| Black HH Adult Ratio | 0.534 | 0.536 | 0.572 | 0.811 |
| Coloured HH Adult Ratio | 0.213 | 0.212 | 0.176 | 0.606 |
| White HH Adult Ratio | 0.031 | 0.029 | 0.04 | 0.704 |
| Female Head Adult Ratio | 0.518 | 0.516 | 0.081 | 0.581 |
| Girls (0-4) in HH | 0.213 | 0.237 | 0.173 | 0.31 |
| Boys (0-4) in HH | 0.209 | 0.228 | 0.234 | 0.649 |
| Girls (5-14) in HH | 0.378 | 0.408 | 0.153 | 0.479 |
| Boys (5-14) in HH | 0.401 | 0.424 | 0.312 | 0.226 |
| Women (15-64) in HH | 1.204 | 1.315 | 0 | 0.03 |
| Men (15-64) in HH | 1.374 | 1.427 | 0.029 | 0.011 |
| Women (65+) in HH | 0.262 | 0.233 | 0.048 | 0.076 |
| Men (65+) in HH | 0.172 | 0.152 | 0.102 | 0.182 |
| Eastern Cape | 0.236 | 0.238 | 0.863 |  |
| Western Cape | 0.093 | 0.098 | 0.57 |  |
| Northern Cape | 0.073 | 0.06 | 0.134 |  |
| Free State | 0.14 | 0.146 | 0.494 |  |
| Kwa-Zulu Natal | 0.066 | 0.071 | 0.541 |  |
| Northwest Province | 0.095 | 0.103 | 0.436 |  |
| Gauteng Province | 0.158 | 0.156 | 0.851 |  |
| Mpumulanga Province | 0.092 | 0.075 | 0.075 |  |
| Urban | 0.777 | 0.765 | 0.349 |  |
| Observations | 1393 | 1393 |  |  |
