# Supplementary material for: The effect of tobacco expenditure on expenditure shares in South African households: A genetic matching approach
Source: PLoS One. 2019 Sep 6;14(9):e0222000. doi: 10.1371/journal.pone.0222000 (PMC6730990; doi:10.1371/journal.pone.0222000)
Supplement: S7 Table — (DOCX) [file pone.0222000.s011.docx]

**S 7 Table. Descriptive statistics before matching for Quartile 4**

| **Variable name** | **Smoking average** | **Non-smoking average** | **t-probability** | **ks-probability** |
| --- | --- | --- | --- | --- |
| Propensity Score | 0.225 | 0.283 | 0 | 0 |
| HH Head Age Group | 10.399 | 10.177 | 0.005 | 0.012 |
| HH Head Schooling | 2.677 | 2.495 | 0 | 0 |
| HH Head Training | 0.245 | 0.258 | 0.316 |  |
| Black HH Head | 0.601 | 0.349 | 0 |  |
| Coloured HH Head | 0.115 | 0.312 | 0 |  |
| White HH Head | 0.284 | 0.339 | 0 |  |
| Female HH Head | 0.674 | 0.759 | 0 |  |
| Black HH Log Inc | 5.668 | 3.281 | 0 | 0 |
| Coloured HH Log Inc | 1.11 | 3.006 | 0 | 0 |
| White HH Log Inc | 2.854 | 3.396 | 0 | 0 |
| Female Head Log Inc | 6.599 | 7.417 | 0 | 0 |
| Log Net Exp | 9.608 | 9.578 | 0.083 | 0.225 |
| Black HH Log Net Exp | 5.679 | 3.273 | 0 | 0 |
| Coloured HH Log Net Exp | 1.096 | 2.949 | 0 | 0 |
| White HH Log Net Exp | 2.833 | 3.357 | 0 | 0 |
| Female Head Log Net Exp | 6.535 | 7.295 | 0 | 0 |
| Black HH Sex Ratio | 0.271 | 0.193 | 0 | 0 |
| Coloured HH Sex Ratio | 0.054 | 0.155 | 0 | 0 |
| White HH Sex Ratio | 0.132 | 0.166 | 0 | 0 |
| Female Head Sex Ratio | 0.382 | 0.438 | 0 | 0 |
| Black HH Adult Ratio | 0.447 | 0.273 | 0 | 0 |
| Coloured HH Adult Ratio | 0.089 | 0.244 | 0 | 0 |
| White HH Adult Ratio | 0.25 | 0.295 | 0 | 0 |
| Female Head Adult Ratio | 0.536 | 0.616 | 0 | 0 |
| Girls (0-4) in HH | 0.163 | 0.155 | 0.551 | 0.811 |
| Boys (0-4) in HH | 0.178 | 0.176 | 0.841 | 0.958 |
| Girls (5-14) in HH | 0.356 | 0.309 | 0.009 | 0.047 |
| Boys (5-14) in HH | 0.353 | 0.328 | 0.188 | 0.124 |
| Women (15-64) in HH | 1.286 | 1.308 | 0.442 | 0.504 |
| Men (15-64) in HH | 1.128 | 1.398 | 0 | 0 |
| Women (65+) in HH | 0.196 | 0.174 | 0.072 | 0.035 |
| Men (65+) in HH | 0.15 | 0.142 | 0.424 | 0.279 |
| Eastern Cape | 0.184 | 0.339 | 0 |  |
| Western Cape | 0.108 | 0.079 | 0.001 |  |
| Northern Cape | 0.049 | 0.055 | 0.381 |  |
| Free State | 0.076 | 0.085 | 0.275 |  |
| Kwa-Zulu Natal | 0.104 | 0.068 | 0 |  |
| Northwest Province | 0.084 | 0.058 | 0 |  |
| Gauteng Province | 0.221 | 0.214 | 0.564 |  |
| Mpumulanga Province | 0.079 | 0.069 | 0.18 |  |
| Urban | 0.843 | 0.921 | 0 |  |
| Observations | 4670 | 1462 |  |  |
