# Supplementary material for: The effect of tobacco expenditure on expenditure shares in South African households: A genetic matching approach
Source: PLoS One. 2019 Sep 6;14(9):e0222000. doi: 10.1371/journal.pone.0222000 (PMC6730990; doi:10.1371/journal.pone.0222000)
Supplement: S8 Table — (DOCX) [file pone.0222000.s012.docx]

**S 8 Table. Descriptive statistics after matching for Quartile 4**

| **Variable name** | **Smoking average** | **Non-smoking average** | **t-probability** | **ks-probability** |
| --- | --- | --- | --- | --- |
| Propensity Score | 0.282 | 0.283 | 0.491 | 0.992 |
| HH Head Age Group | 10.358 | 10.176 | 0.028 | 0.031 |
| HH Head Schooling | 2.497 | 2.495 | 0.78 | 0.804 |
| HH Head Training | 0.256 | 0.258 | 0.872 |  |
| Black HH Head | 0.349 | 0.349 | 1 |  |
| Coloured HH Head | 0.312 | 0.312 | 1 |  |
| White HH Head | 0.339 | 0.339 | 1 |  |
| Female HH Head | 0.759 | 0.759 | 1 |  |
| Black HH Log Inc | 3.275 | 3.281 | 0.454 | 0.685 |
| Coloured HH Log Inc | 3.003 | 3.006 | 0.819 | 0.558 |
| White HH Log Inc | 3.406 | 3.396 | 0.254 | 0.702 |
| Female Head Log Inc | 7.425 | 7.417 | 0.485 | 0.406 |
| Log Net Exp | 9.576 | 9.578 | 0.417 | 0.751 |
| Black HH Log Net Exp | 3.272 | 3.273 | 0.402 | 0.818 |
| Coloured HH Log Net Exp | 2.947 | 2.949 | 0.437 | 0.825 |
| White HH Log Net Exp | 3.357 | 3.356 | 0.864 | 0.985 |
| Female Head Log Net Exp | 7.295 | 7.295 | 0.874 | 0.901 |
| Black HH Sex Ratio | 0.193 | 0.193 | 0.892 | 0.845 |
| Coloured HH Sex Ratio | 0.156 | 0.155 | 0.453 | 0.867 |
| White HH Sex Ratio | 0.164 | 0.166 | 0.351 | 0.791 |
| Female Head Sex Ratio | 0.438 | 0.437 | 0.775 | 0.264 |
| Black HH Adult Ratio | 0.272 | 0.273 | 0.494 | 0.844 |
| Coloured HH Adult Ratio | 0.246 | 0.244 | 0.434 | 0.823 |
| White HH Adult Ratio | 0.296 | 0.295 | 0.251 | 0.982 |
| Female Head Adult Ratio | 0.618 | 0.616 | 0.454 | 0.896 |
| Girls (0-4) in HH | 0.171 | 0.155 | 0.265 | 0.311 |
| Boys (0-4) in HH | 0.159 | 0.176 | 0.235 | 0.211 |
| Girls (5-14) in HH | 0.269 | 0.309 | 0.028 | 0.101 |
| Boys (5-14) in HH | 0.334 | 0.328 | 0.744 | 0.116 |
| Women (15-64) in HH | 1.244 | 1.308 | 0.005 | 0.262 |
| Men (15-64) in HH | 1.341 | 1.398 | 0.009 | 0.03 |
| Women (65+) in HH | 0.208 | 0.174 | 0.016 | 0.008 |
| Men (65+) in HH | 0.18 | 0.142 | 0.002 | 0.011 |
| Eastern Cape | 0.369 | 0.339 | 0 |  |
| Western Cape | 0.1 | 0.079 | 0.03 |  |
| Northern Cape | 0.062 | 0.055 | 0.361 |  |
| Free State | 0.061 | 0.085 | 0.008 |  |
| Kwa-Zulu Natal | 0.06 | 0.068 | 0.314 |  |
| Northwest Province | 0.047 | 0.057 | 0.13 |  |
| Gauteng Province | 0.209 | 0.214 | 0.676 |  |
| Mpumulanga Province | 0.061 | 0.069 | 0.36 |  |
| Urban | 0.923 | 0.921 | 0.724 |  |
| Observations | 1462 | 1462 |  |  |
